# The origin of blinking in both mudskippers and tetrapods is linked to life on land

**DOI:** 10.1073/pnas.2220404120

**Published:** 2023-04-24

**Authors:** Brett R. Aiello, M. Saad Bhamla, Jeff Gau, John G. L. Morris, Kenji Bomar, Shashwati da Cunha, Harrison Fu, Julia Laws, Hajime Minoguchi, Manognya Sripathi, Kendra Washington, Gabriella Wong, Neil H. Shubin, Simon Sponberg, Thomas A. Stewart

**Affiliations:** ^a^Department of Biology, Seton Hill University, Greensburg, PA 15601; ^b^School of Physics, Georgia Institute of Technology, Atlanta, GA 30332; ^c^School of Biological Science, Georgia Institute of Technology, Atlanta, GA 30332; ^d^Living Dynamical Systems Vertically Integrated Project Team, Georgia Institute of Technology, Atlanta, GA 30332; ^e^School of Chemical and Biomolecular Engineering, Georgia Institute of Technology, Atlanta, GA 30332; ^f^Interdisciplinary Bioengineering Graduate Program, Georgia Institute of Technology, Atlanta, GA 30332; ^g^George W. Woodruff School of Mechanical Engineering, Georgia Institute of Technology, Atlanta, GA 30332; ^h^Department of Neurology, Westmead Hospital, Sydney, NSW 2145, Australia; ^i^Department Organismal Biology and Anatomy, The University of Chicago, Chicago, IL 60637; ^j^Department of Biology, The Pennsylvania State University, State College, PA 16802

**Keywords:** vision, evolutionary novelty, water-to-land transition

## Abstract

Blinking, the transient occlusion of the eye by one or more membranes, serves several functions including wetting, protecting, and cleaning the eye. This behavior is seen in nearly all living tetrapods and absent in other extant sarcopterygian lineages suggesting that it might have arisen during the water-to-land transition. Unfortunately, our understanding of the origin of blinking has been limited by a lack of known anatomical correlates of the behavior in the fossil record and a paucity of comparative functional studies. To understand how and why blinking originates, we leverage mudskippers (Oxudercinae), a clade of amphibious fishes that have convergently evolved blinking. Using microcomputed tomography and histology, we analyzed two mudskipper species, *Periophthalmus barbarus* and *Periophthalmodon septemradiatus*, and compared them to the fully aquatic round goby, *Neogobius melanostomus*. Study of gross anatomy and epithelial microstructure shows that mudskippers have not evolved novel musculature or glands to blink. Behavioral analyses show the blinks of mudskippers are functionally convergent with those of tetrapods: *P. barbarus* blinks more often under high-evaporation conditions to wet the eye, a blink reflex protects the eye from physical insult, and a single blink can fully clean the cornea of particulates. Thus, eye retraction in concert with a passive occlusal membrane can achieve functions associated with life on land. Osteological correlates of eye retraction are present in the earliest limbed vertebrates, suggesting blinking capability. In both mudskippers and tetrapods, therefore, the origin of this multifunctional innovation is likely explained by selection for increasingly terrestrial lifestyles.

## Significance

Most tetrapods blink, closing their eyes periodically with eyelids or a nictitating membrane, and blinking is critical for maintaining eye health. In humans, for example, the inability to blink regularly can lead to vision loss. However, how and why did blinking first evolve? It has been difficult to tackle this question from the fossil record alone. This study sheds light on the origin of blinking by considering a second lineage of fishes that have convergently evolved blinking behaviors: the mudskippers. By analyzing how blinking behaviors are performed and testing hypotheses of blink function in mudskippers, we show how anatomical systems can be tinkered with to achieve a novel behavior and argue that blinking is an adaptation to life on land.

**Fig. 1.**
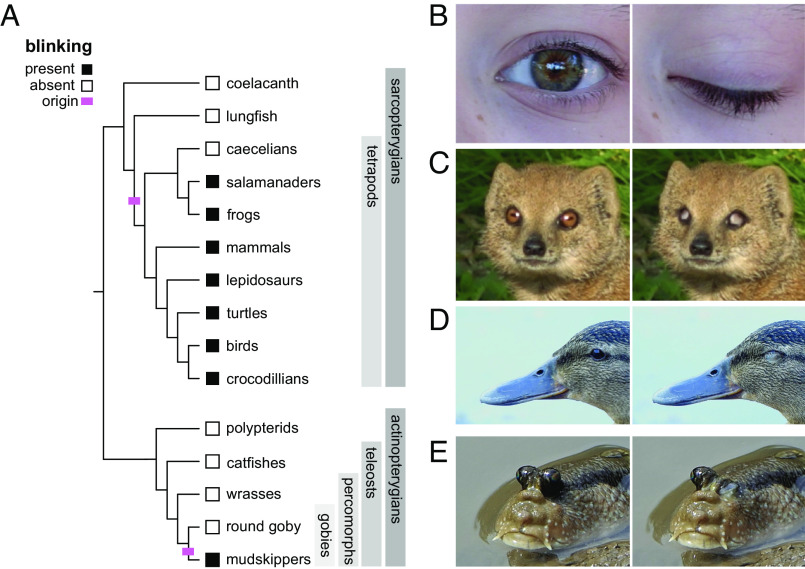
Blinking has evolved repeatedly. (*A*) A phylogeny showing the distribution of blinking in osteichthyans (bony fishes). A black square indicates that blinking is present within a clade. A white square indicates that blinking is absent in the group. A pink hash mark indicates where blinking is hypothesized to have originated. In some clades labeled with black squares, where blinking generally characterizes the group, blinking has been secondarily lost (e.g., snakes and some fully aquatic frogs). Among actinopterygians, select clades are shown to convey both the general condition among the more than 30,000 actinopterygians, the absence of blinking, as well as the phylogenetic position of mudskippers. (*B*) In humans, blinking involves lowering of the upper eyelid. Other tetrapods, however, can blink with other membranes. (*C*) A yellow mongoose (*Cynictis penicillata*) closing its eyes with a nictitating membrane, which moves in a rostrocaudal direction. (*D*) A mallard duck (*Anas platyrhynchos*) closing its eye by raising the lower eyelid. (*E*) A pug-headed mudskipper (*Periophthalmodon freycineti*) blinking and showing movement of the eye and dermal cup. All images by John Morris.

Approximately 375 Mya, stem-group tetrapods transitioned from life in the water to life on land (1–3). This transition involved a suite of anatomical transformations, including modifications to the feeding, locomotor, and sensory systems (4–12). Blinking, a behavior in which one or more membranes transiently occlude the eye, occurs in all major crown group tetrapod lineages and is absent in closely related, aquatic lineages (i.e., coelacanth and lungfish) (Fig. 1*A*) (13, 14). Its origin, therefore, might have coincided with the water-to-land transition. However, the lack of fossilization of associated morphologies (e.g., eyelids and lacrimal glands) and a lack of comparative functional analyses limit hypotheses of how and why blinking first evolved. Several other lineages of fishes have evolved to live at the water’s edge (15–18). Analyses of these groups might reveal both the anatomy required to perform a blink and the selective pressures that lead to the origin of this behavior.

In tetrapods, the anatomy associated with blinking is diverse (13, 14). For example, there is variation in which and how many membranes occlude the eye. Whereas humans largely blink by lowering of the upper eyelid, blinking can also be achieved by elevating of the lower eyelid or moving a nictitating membrane posterolaterally (Fig. 1 *B*–*D*). The movement of these membranes can result directly from the activation of eyelid-associated musculature or indirectly by eye retraction (19–23). Tetrapods also have glands that secrete tears, the aqueous and oily liquid that forms a film on the surface of the cornea (24). The identity of the multicellular secretory glands that are present in the orbit and eyelid (e.g., harderian, lacrimal and meibomian glands) can differ between tetrapod lineages (14, 25, 26). The variation in anatomical features associated with blinking in living tetrapods hinders inferences of ancestral state and, thus, determination of which structures evolved to first produce this behavior. However, osteological correlates on the skull of early limbed vertebrates (e.g., *Acanthostega gunnari*) suggest the presence of a retractor bulbi muscle (27) and, perhaps, the ability for closure by eye retraction.

The diverse morphologies associated with blinking are paralleled by the diversity of functions that blinking serves in tetrapods (13, 28–33). Blinking usually occurs as an endogenous, or spontaneous, behavior that covers the cornea in a fluid film (13, 24, 31, 34, 35). This fluid layer allows for the transfer of oxygen from air to cells in the corneal epithelium (36, 37), which is not vascularized (14, 38, 39). Further support that blinking functions to wet the corneal surface comes from humans, the species in which blinking has been studied most extensively. In humans, blink frequency is inversely related to air humidity (28). Blinking can also be induced by exogenous stimuli. For example, a blink can be prompted by the encroachment of objects that pose the risk of injury or by debris that might scratch the cornea or otherwise obstruct vision (14, 28). Exogenous blinks, which can be reflexive, often involve a faster rate of eye closure than endogenous blinks (40, 41). Limited comparative functional data make it difficult to discriminate between potential selective pressures, such as wetting, cleaning, or protecting the eye, that might have contributed to the origin of blinking in stem tetrapods.

Several lineages of fishes have independently evolved to live at the interface between water and land, and they can inform how and why blinking originates. Mudskippers (Gobiidae, Oxudercinae) are among the charismatic examples of amphibious fishes. They are found in mangroves and tidal flats of the Indo-West Pacific the eastern Atlantic oceans, spending a significant portion of their day on land as adults (17, 42–45). Among the adaptations that characterize this clade is a blinking behavior (Fig. 1*E*). When mudskippers blink, they lower their eyes, which sit high upon the head, while a membrane called the dermal cup rises to occlude the cornea (43, 46–48). Other gobies do not exhibit this behavior or possess the associated morphologies (i.e., elevated eyes and dermal cup) (49). Although the function of mudskipper blinking is unknown, several hypotheses have been proposed: to moisten the eye while on land (50), to protect the eye from injury by encroaching objects (43), or to clean the eye of debris (46, 50). The goal of this study is first to analyze the morphological basis of blinking in mudskippers and second to test hypotheses of its function. Elucidating how and why blinking originated in these fishes might help to clarify the biology of early tetrapods and their transition to life on land.

## Results

### Endogenous Blinking Is a Terrestrial Behavior.

The Indian mudskipper, *Periophthalmodon septemradiatus,* and the African mudskipper, *Periophthalmus barbarus,* regularly blink on land, both bilaterally and unilaterally (Movies S1–S3). Adult individuals of each species were housed in tanks that allowed for free movement between water and land. Although blinking has been noted in mudskippers underwater (46), we did not observe endogenous blinking underwater. In our study, blinking was observed only rarely underwater, when individuals bumped their heads on objects in the tank (e.g., water filter, other individuals).

### Mudskippers Blink by Lowering the Eye and Raising a Membrane.

**Fig. 2.**
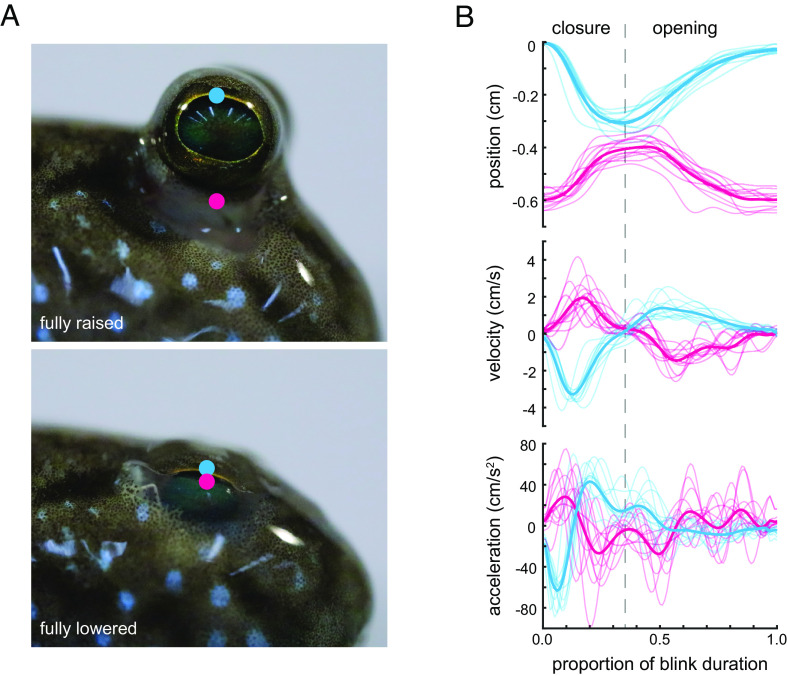
Mudskippers blink spontaneously on land. (*A*) Exemplar images of a blink in the Indian mudskipper *P. septemradiatus*. *Top* image shows the eye raised, at rest. *Bottom* image shows the eye fully lowered. Footage of spontaneous mudskipper blinks are shown in Movies S1–S3. (*B*) Kinematics were analyzed in the African mudskipper *P. barbarus.* As illustrated in panel *A*, landmarks were placed on the most dorsal aspect of the corneal surface (blue) and on the most dorsal aspect of the dermal cup (pink). Graphs show lateral kinematics from four individuals (n = 3 blinks per individual, n = 12 total blinks). Individual blinks are plotted as light-colored lines. Mean kinematic trajectories are plotted as bold lines. The start of a blink was defined as when eye depression is first observed. The end of the blink was defined as when the eye returned to its starting point or when velocity of eye elevation reached zero, as the eye would often come to rest at a position lower than where the blink started. The transition from eye closing to eye opening was defined as the point when the eye began to elevate.

Consistent with previous reports, videography shows that mudskippers blink by ventrally retracting the eye into an infraorbital space in conjunction with dorsal elevation of the dermal cup membrane (Fig. 2*A* and Movies S1–S3). We collected three-dimensional blinking kinematics in four individuals of *P. barbarus* (12 blinks total, three blinks per individual). Eye movement was found to be primarily restricted to the dorsoventral axis, and therefore our analyses consider movement in this direction. The duration of spontaneous blinks ranged from 480 to 700 ms with a mean duration of 560 ± 73 ms (mean ± SD) (*SI Appendix*, Table S1). This duration is approximately the same as the spontaneous blink of humans, which is 572 ± 25 ms (51). Movement of the mudskipper dermal cup starts slightly after the onset of eye movement, at around 2% of the total blink duration, and it achieves maximum elevation coincident with full eye depression, at 35 ± 3% of total blink duration (Fig. 2*B* and *SI Appendix*, Table S1). Peak eye velocity and acceleration are significantly higher during depression than during elevation (*P* < 10^−19^) (*SI Appendix*, Table S1). An asymmetry in velocity of eye closing is also observed in all tetrapods for which data is available, with eye closure similarly being faster than eye opening (41, 51–54).

### No Novel Musculature is Associated with Blinking in Mudskippers.

**Fig. 3.**
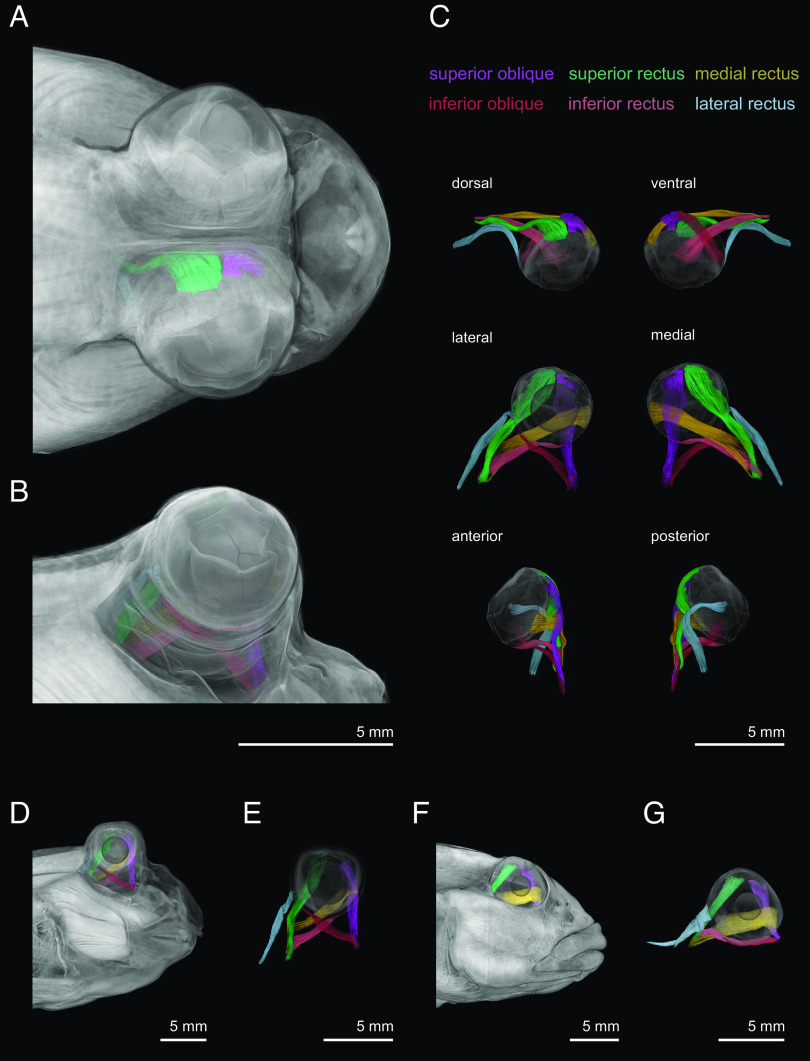
Mudskippers have not evolved novel eye musculature. Volumetric renderings of contrast-enhanced μCT scans show that mudskippers have retained the plesiomorphic set of eye-associated muscles. The same six extraocular muscles are observed in (*A*–*C*) the African mudskipper *P. barbarus*, (*D* and *E*) the Indian mudskipper *P. septemradiatus*, and (*F* and *G*) the fully aquatic round goby, *N. melanostomus*.

To understand how mudskippers blink, we next analyzed cranial morphology and found that mudskippers have not evolved novel musculature associated with either the eye or surrounding tissues. We compared *P. barbarus* and *P. septemradiatus* to the round goby, *Neogobius melanostomus*, which represents a generalized, fully aquatic goby morphology. We stained the specimens with phosphomoloybdic acid to enhance contrast between soft tissues and microcomputed tomography (µCT) scanned. We observed that all three species possess the six extraocular muscles that are apomorphic of jawed vertebrates (Fig. 3 and Movie S4). This observation is consistent with previous gross dissections of mudskippers (55–57). The lack of novel musculature attaching to the eye or to the dermal cup shows that mudskipper eye retraction is driven entirely by some combination of the extraocular muscles and that dermal cup movement is passive. Dermal cup elevation, therefore, likely occurs due to its displacement caused by eye depression, which results in the dermal cup membrane stretching laterally, until it passively vertically slides over the dorsal half of the eye.

Therefore, the origin of blinking in mudskippers reflects rearrangement of the lines of action of a plesiomorphic set of muscles coupled with the evolution of a novel occlusal membrane. Although there are minor differences in the positions of attachment of the extraocular muscles to the eye (e.g., mudskippers have the inferior oblique and inferior rectus more dorsally positioned on the eye compared to the round goby), the major shift in the orientation of extraocular muscles in mudskippers results from the eye being vaulted dorsally (Fig. 3 and *SI Appendix*, Fig. S1). Based on the lines of action of the extraocular muscles, we predict that eye retraction is driven primarily by cocontraction of the superior oblique and the superior rectus, which are the most vertically orientated of these muscles (Fig. 3 and *SI Appendix*, Fig. S1). Testing is required to determine possible contributions by the other extraocular muscles because eye-retraction in tetrapods can involve cocontraction of up to all six of the extraocular muscles, as in turtles (21). The positioning of the extraocular muscles additionally suggests that eye opening could be a passive process. In mudskippers, all six of these muscles attach to the cranium in a position ventral to the eye, suggesting that the dorsal elevation of the eye is not driven by direct action by these muscles (Fig. 3 and *SI Appendix*, Fig. S1). The elastic recoil of the dermal cup, which is stretched and displaced during eye depression, might contribute to eye raising. The active generation of buccal pressure could also contribute to eye elevation, as in some amphibians (58). Regardless, the musculature necessary for blinking in mudskippers reflects variation upon an existing suite of muscles, rather than origination of novel actuation elements.

### No Novel Multicellular Glands Are Associated with Blinking in Mudskippers.

**Fig. 4.**
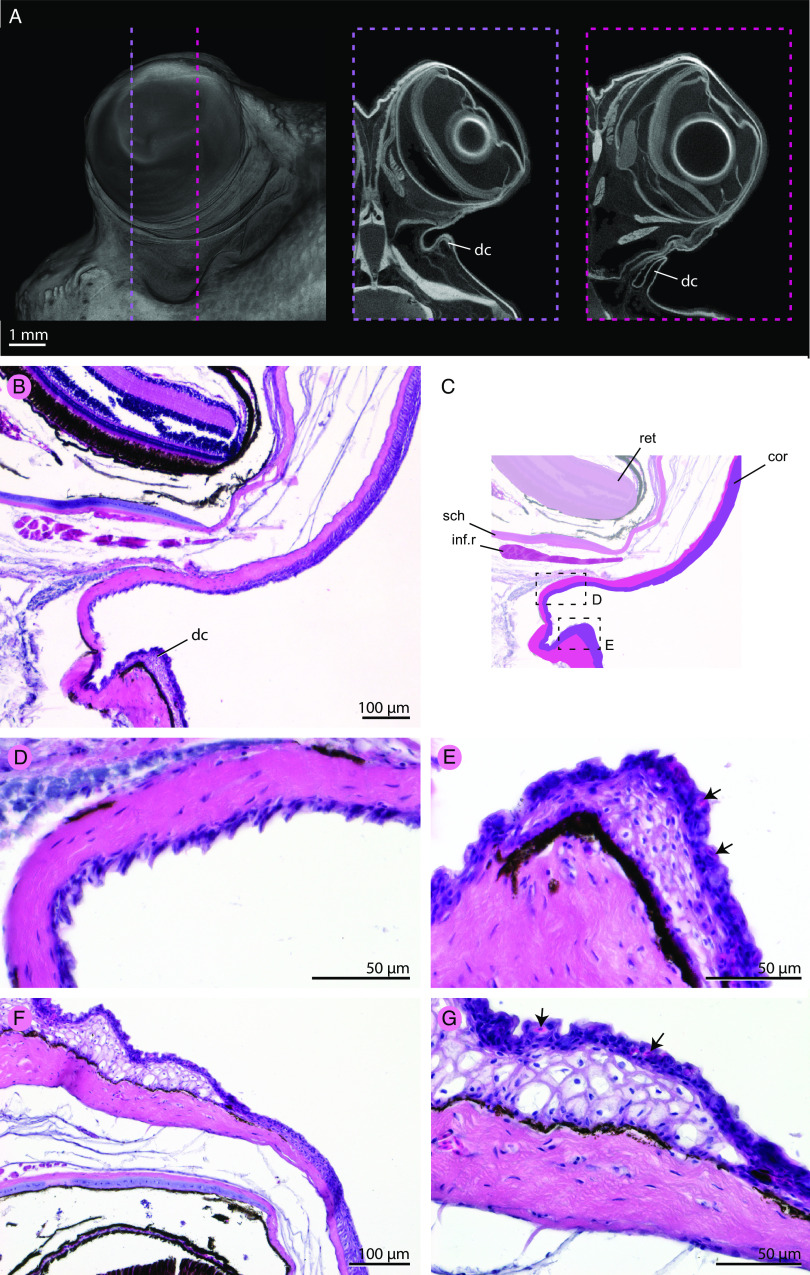
Mudskippers have not evolved novel “tear glands.” (*A*) Volumetric rendering of contrast-enhanced μCT scan of *P. barbarus* showing left lateral view of the head. The dashed purple and pink lines show the position of digital cross-sections, to the right, which were studied to assess whether multicellular glands or associated ducts were present around the eye; none were observed. (*B*–*G*) Parasagittal sections the eye of *P. septemradiatus* and surrounding tissues stained with hematoxylin and eosin to confirm observations from µCT data. Panel *B* is of the same position as the purple dashed line of panel *A*. Panel *C* labels anatomical features of this section and shows regions that are zoomed in on panels *D* and *E*. Panel *D* shows distinct microstructure of the epithelium below the eye. (*F*) Epithelium dorsal to the eye at the same sagittal section as panels *B*–*E*. (*G*) A higher magnification of the tissue in panel *F* that shows the organization of the epithelium dorsal to the eye. Arrows in panels *E* and *G* denote putative secretory cells. Abbreviations: cor, cornea; inf.r, inferior rectus; ret, retina; sch, schlera.

To test whether mudskippers have evolved multicellular glands around the orbit to produce a tear film, convergent with tetrapods, we analyzed µCT data and histology. In tetrapods, the tear film is produced by glands around the orbit or in the eyelids (e.g., harderian, lacrimal, meibomian glands); the anatomy of these glands has been previously characterized histologically (59–63). We found no evidence of multicellular glands or ducts (structures associated with multicellular glands) around the eye or in association with the dermal cup in the µCT data of *P. barbarus* and *P. septemradiatus* (Fig. 4*A* and Movie S5). This is consistent with previous histological work on *P. barbarus* (64), which did not describe multicellular glands around the eye. We further validated these results with histology in *P. septemradiatus* (Fig. 4 *B*–*G*). However, histological sections revealed cells in the epithelium of the dermal cup that we diagnose as secretory cells based on their shape, position, and differential staining relative to other epithelial cells (Fig. 4*E*) (65). These secretory cells are likely goblet cells, which produce the mucus of fish skin. We find that these cells are not spatially concentrated around the eye but distributed in similar concentrations as in other epithelia on the cranium (Fig. 4*E*). Therefore, we predict that if blinking functions to wet the eyes of mudskippers, the fluids being spread include mucus from the epithelium of the dermal cup and surrounding tissues on the head.

As with musculature, mudskippers show no evidence of novel “tear” glands in their cranial anatomy. Given the distinct anatomical organization for blinking in mudskippers compared to tetrapods, we next tested whether blinking is, in fact, functionally convergent between mudskippers and tetrapods.

### Blinking Serves to Wet the Eye.

**Fig. 5.**
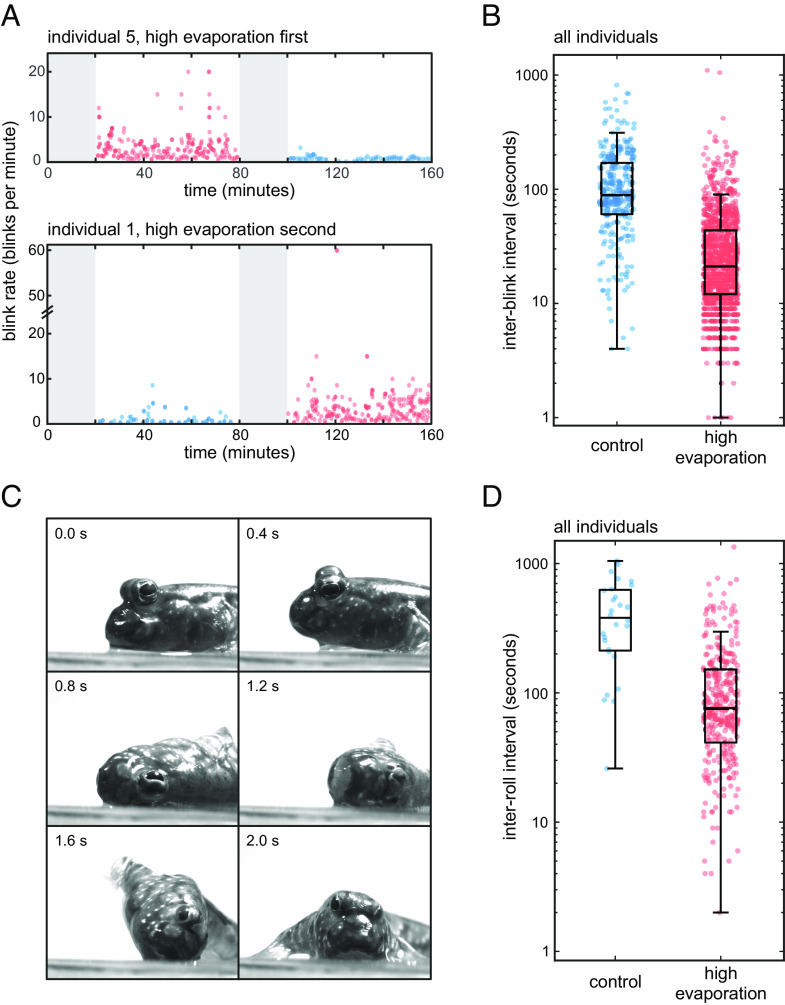
Blink rate and roll rate increase under high-evaporation conditions. (*A*) *P. barbarus* were exposed to control and high-evaporation conditions and blink rate was recorded. Data are presented from two such experiments that differed in treatment order (i.e., high-evaporation condition first or second). Blink rate was measured for each eye individually. Left and right eyes do not show differences, and so data from both eyes were pooled. (*B*) Interblink interval (IBI), the reciprocal of instantaneous blink rate, is significantly shorter under high-evaporation conditions than under control conditions. (*C*) Mudskippers exhibit a behavior where the body rolls laterally around its long axis while on land. Images of *P. barbarus* rolling. Footage of rolling behavior is shown in Movie S6. (*D*) *P. barbarus* interroll interval (IRI) was compared between control and high-evaporation treatments. IRI is significantly shorter under high-evaporation conditions. Associated statistics for panels *B* and *D* are shown in *SI Appendix*, Table S3. Boxplots show the median value, the 25th and 75th percentiles, and the range of data points not considered to be outliers.

To test the functional hypothesis that mudskipper blinking functions to wet the cornea, we investigated whether evaporation rate affects the frequency of spontaneous blinking. We filmed mudskippers were under two treatments, ambient room and high-evaporation conditions. We varied which condition was presented first during experimentation between individuals (Fig. 5*A*). To create the high-evaporation condition, we increased the air flow through the tank, which resulted in an evaporation rate approximately 30 times greater than in ambient control conditions (see *Materials and Methods* for calculation of evaporation rate). Under the high-evaporation condition, *P. barbarus* showed a significantly lower interblink interval (IBI), or the time between successive blinks of an eye, as compared to control (*P *< 0.0001) (Fig. 5*B* and *SI Appendix*, Table S2). These results are consistent with the hypothesis that eye wetting is one function of mudskipper blinking.

We observed that *P. barbarus* performed a whole-body rolling behavior while on land. The animal rotates around the long axis of the body, placing one side of its head adjacent to the ground (Fig. 5*C* and Movie S6). We hypothesized that this rolling behavior is a means of wetting the body by capturing moisture from the environment. During the roll, the eye is retracted, and the cornea is covered by the dermal cup such that when the head is positioned against the ground laterally the dermal cup membrane is in contact with fluids on the ground (e.g., tank water) (Movie S6). The eyes raise and the dermal cup retracts when the roll is complete. This behavior could capture water to wet the eye and is consistent with past speculation that environmental water is held in a reservoir in the infraorbital space behind the dermal cup membrane (46–48). To test the prediction that the whole-body rolling behavior is impacted by evaporation rate, we quantified the interroll interval (IRI), or the time between successive rolls, under control and high-evaporation conditions. Under control conditions, two of the six individuals did not roll, and one individual only rolled a single time during the 1-h filming period under typical ambient conditions. For the three individuals that repeatedly rolled, the IRI was 428.1 ± 279.9 s (Fig. 5*D* and *SI Appendix*, Table S2). Under high-evaporation conditions, all six individuals performed the rolling behavior and the IRI was significantly shorter: 127.1 ± 147.5 s (*P *< 0.0001) (Fig. 5*D* and *SI Appendix*, Table S2). These results are consistent with the hypotheses that mudskipper rolling is a response to drying and that the animals use environmental fluids, in this case the water on the bottom of the tank, to moisten the body and eye, possibly by capturing it in the infraorbital space behind the dermal cup membrane (43, 46, 47). Therefore, we predict that the fluid film on the cornea of mudskippers is a combination of secretions from broadly distributed mucus glands on the head and water from the environment.

### Blinking Cleans the Corneal Surface.

**Fig. 6.**
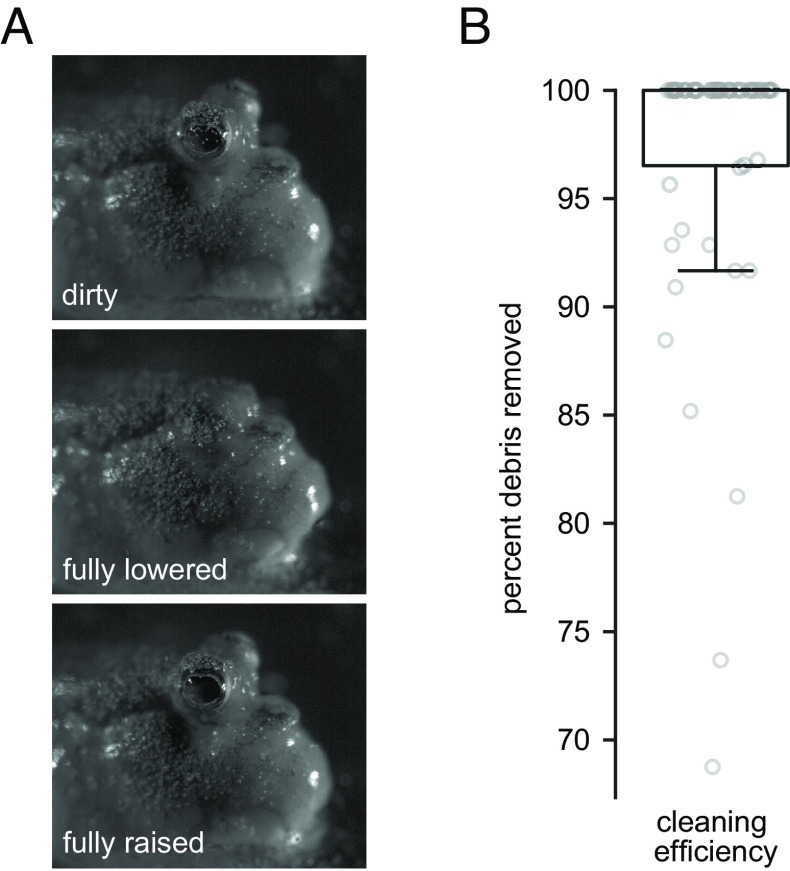
Blinking cleans the cornea. (*A*) The heads of *P. barbarus* were sprinkled with brine shrimp eggs. Blinking removes debris from the cornea. Footage of cleaning behavior is shown in Movie S7. (*B*) Percentage of brine shrimp eggs removed from the cornea in a single blink. Boxplot shows the median value, the 25th and 75th percentiles, the range of data points not considered to be outliers.

To test the hypothesis that mudskipper blinking functions to clean the corneal surface, we dusted dry brine shrimp eggs onto the eyes of *P. barbarus*. We selected these particles because their size (diameter ~200 µm) is similar to sand (66), a material regularly encountered in the natural environment of mudskippers. We applied approximately 15 particles to the eye per trial. *P. barbarus* was able to remove almost all particles from the cornea in a single blink. We defined cleaning efficiency as the percentage of particles removed from an eye in a single blink. Across all five individuals (n = 53 trials), cleaning efficiency was 97 ± 7% (Fig. 6, Movie S7, and *SI Appendix*, Table S3).

### Blinking Serves to Protect the Eye.

**Fig. 7.**
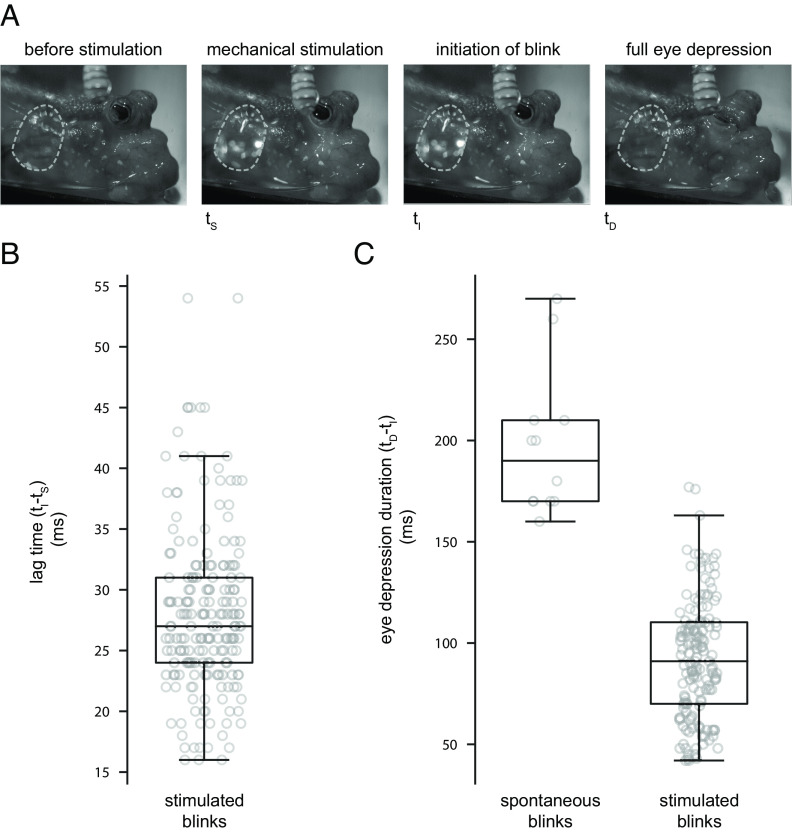
Mechanical stimulation of the cornea initiates a blink reflex. (*A*) In *P. barbarus,* the posterodorsal portion of the cornea was gently touched with a capacitance probe that, when in physical contact with the eye, illuminates an LED light (outlined by dashed line). Touching the eye caused a blink. Footage of blinks caused by mechanical stimulation is shown in Movie S8. (*B*) The lag time between corneal stimulation and the initiation of the blink is on average less than 30 ms. (*C*) Duration of the depression phase of the blink cycle is significantly shorter in mechanically induced exogenous blinks than spontaneous blinks. Associated statistics presented in *SI Appendix*, Table S4. Boxplots show the median value, the 25th and 75th percentiles, the range of data points not considered to be outliers.

To test the hypothesis that mudskipper blinking functions to protect the eye from injury by large-scale objects, we investigated whether mechanical stimulation of the cornea elicits a blink response. We used a soft capacitance probe, which illuminated a light-emitting diode when in contact with the skin, to mechanically stimulate the eye and quantify blink lag time (i.e., time between mechanical stimulation and the start of the blink) (Fig. 7*A* and Movie S8). Across all five individuals (n = 188 trials), the lag time was 28 ± 7 ms (Fig. 7*B*). Duration is similar to the lag time of the corneal reflex in humans (25 to 40 ms), in which a blink is induced by mechanical stimulation of the cornea (40, 67). The duration of eye depression in mechanically stimulated blinks in the mudskipper was 93 ± 30 ms (*SI Appendix*, Table S4), significantly shorter than endogenous blinks (*P *< 0.0001) (Fig. 7*C*). This pattern also mirrors humans, where the duration of eye closure in corneal reflexive blinks is shorter than endogenous blinks (41).

Comparisons of blinks caused by mechanical stimulation of the cornea and endogenous blinks reveal similarities between mudskippers and humans. Both species have similar lag time durations and shorter eye closure durations when the blink is mechanically stimulated in comparison to endogenous blinks. These similarities suggest that mudskippers have evolved a reflex that is convergent to the corneal reflex of tetrapods.

## Discussion

Mudskippers have evolved a blinking behavior in which the eye retracts ventrally, actuated by some combination of the six extraocular muscles that are plesiomorphic to jawed vertebrates. This causes a membrane, the dermal cup, to be displaced and passively move upward to occlude the eye. Comparative analyses show that no novel musculature, multicellular glands, or higher concentrations of secretory cells have evolved for this behavior. Nevertheless, it is functionally convergent with the blinks of tetrapods, capable of wetting, cleaning, and protecting the eye. In both mudskippers and tetrapods, blinking appears to have originated coincident with terrestriality. We predict that similar selective demands have acted upon the visual systems of these lineages and propose that blinking is a key adaptation that has facilitated for a major shift in niche occupancy and life on land.

Although functionally convergent, the blinks of mudskippers likely rely on a distinct sensorimotor architecture as compared to tetrapods. In both clades, sensory innervation from the cornea likely travels by the trigeminal nerve; this is well established across tetrapods (19, 20, 68, 69) and appears to be conserved across osteichthyans (70). However, the motor innervation in mudskippers likely involves some combination of the oculomotor, abducens, or trochlear nerves, which are conserved in innervating the extraocular muscles across jawed vertebrates (23). In tetrapods, eye closure is usually achieved by the direct actuation of eyelids or nictitating membrane (13, 14), and the muscles that drive these movements are innervated by the facial nerve (23). Blinking in tetrapods can also involve eye retraction by the retractor bulbi (e.g., cats, rabbits, and turtles) (19–21), which is innervated by the abducens (23) and oculomotor nerves (71). These differences in the physiological basis of blinking of mudskippers and tetrapods show the potential for multiple evolutionary solutions to the origination of complex, multifunctional behaviors that arise during major ecological transitions.

### Hypotheses for the Origin of Blinking in Mudskippers.

Blinking might originate as an aquatic behavior that is secondarily exapted for terrestrial environments. In fully aquatic fishes, analogous behaviors involving retraction of the eye into a retrobulbar space that results in transient occlusion of the cornea by conjunctiva have evolved independently at least three times (i.e., guitar fish, whale sharks, and puffer fish) (72–74). Additionally, some chondrichthyans can transiently cover their eyes with a novel nictitating membrane (75). In both cases, whether by eye withdrawal or by actively moving a membrane, eye coverage is hypothesized to provide protection from physical insult by objects in the water (72, 73, 75). It is possible that blinking in mudskippers and tetrapods could have arisen for similar reasons: first for protection underwater with additional functions, like wetting and cleaning, being secondarily selected for or evolving as side effects during terrestrialization.

Alternatively, blinking might originate in response to the selective pressures associated with life on land. The challenge of oxygenating corneal cells, for example, is markedly different between aquatic and aerial conditions. The cornea lacks vasculature for optical clarity, and corneal epithelial cells receive oxygen by diffusion from the environment (14, 36, 38). Diffusion occurs more readily in aquatic environments and across wet surfaces exposed to air as compared to across dry surfaces (37). Both tetrapods and mudskippers appear to have circumvented the consequences of diminished gas exchange on dry eyes with endogenous blinking, which maintains a fluid film layer on the cornea. Additionally, the challenge of keeping the cornea clean also differs between aquatic and terrestrial environments. For example, the tendency of detritus to adhere to the cornea, which could obstruct vision or scratch the eye, is different in water and on land. The propensity for or the nature of eye injuries might also differ between these environments. For example, it is likely that the velocity at which objects of comparable size approach an eye will be greater in air than in water, because of the approximately 50-fold difference in viscosity between these liquids (76).

In mudskippers, we predict that blinking first evolved as a terrestrial behavior. We do not find evidence of the behavior or associated morphology in taxonomic outgroups and, thus, no phylogenetic evidence for an aquatic origin. Further, in mudskippers there is a tight correlation between life history and anatomy. Juvenile mudskippers are fully aquatic, and their eyes are positioned like other gobies, not elevated (77). It is only upon metamorphosis, when they begin to leave the water, that the eyes elevate (77); we predict that this is when in ontogeny blinking begins. These observations suggest that a scenario where blinking in mudskippers originated for a protective function in an aquatic context is unlikely.

Our results show that mudskippers blink on land for multiple distinct reasons and do not lead us to identify one function above the others as uniquely causative in its evolutionary origin. Perhaps the multiple and independent functions associated with aerial vision–wetting, cleaning, and protection–originated as a suite, instead of as a stepwise process where an incipient function (e.g., protection) evolved first and other functions evolved secondarily or as side effects. Indeed, a confluence of multiple selective pressures acting concurrently might explain why this complex behavior evolved convergently in lineages separated by many millions of years.

### The Origin of Blinking in Tetrapods.

In tetrapods, it is likely that blinking also originated during the water-to-land transition. The eyes of fully aquatic stem group tetrapods, like *Eusthenopteron foordi*, are positioned laterally on the skull. In more crownward taxa that are inferred to have lived at the water’s edge and perhaps made brief forays onto land (e.g., *Tiktaalik roseae*, *Elpistostege watsoni*, and *Parmastega aelidae*), the eyes are dorsally positioned on the cranium, slightly raised, and with bony brows (10, 12, 78). Several of these species show proportionally enlarged eyes as compared to the plesiomorphic condition, suggesting selection for aerial vision (11). Further crownward, *A. gunnari*, one of the earliest limbed taxa, shows perhaps the first direct evidence of eye closure, with a bilateral pair of depressions on the neurocranium indicative of the attachment site for the retractor bulbi (27). Thus, the paleontological record suggests that blinking in tetrapods originated after aerial vision by an eye retraction mechanism, with the eye being withdrawn medioventrally and occluded by surrounding conjunctiva or epithelial folds. It is likely that blinking in tetrapods did not originate as an aquatic behavior, but instead was a consequence of selection for aerial vision and increasingly terrestrial lifestyles.

A corollary of this scenario for the origin of blinking in tetrapods is that the variation in anatomical features associated with blinking in living species reflects a secondary elaboration and diversification of this system. If, as the anatomy of *A. gunnari* implies, the tetrapod blink originated through an eye retraction by the retractor bulbi, then evolution of actively controlled eyelids occurred after the origin of blinking, and variation in neural architecture of the corneal reflex seen in extant tetrapods reflects lability of reflex arc motor innervation. As mudskippers show, hypotheses for blink origin in tetrapods are not dependent upon the timing of the origin of tear glands. However, in mudskippers, the use of environmental moisture to wet the eye likely reflects a constraint on sustaining extended periods of time away from water. The evolution of specialized tear glands in tetrapods could ultimately have been key in allowing for extended periods of time away from environmental sources of wetting and for individuals to make sustained incursions into terrestrial habits.

The convergent evolution of blinking in two vertebrate lineages that diverged approximately 425 Mya (79) is a reflection of both adaptation and constraint (80). Each group independently underwent a transition to living on land, and the emergence of this innovation is a consequence of shared selective pressures producing a complex, multifunctional behavior. However, it is also the commonalities in the anatomy and physiology of their eyes that resulted in analogous solutions to the functional challenges of leaving water.

## Materials and Methods

### Experimental Design.

First, the blinking behavior was observed in the African mudskipper *Periophthalmus barbarus* and the Indian mudskipper *P. septemradiatus*. Next, μCT scanning was performed on these two mudskipper species and the round goby, *N. melanostomus*. Histological analysis was performed in *P*. *septemradiatus*; histological data had been previously collected for *P*. *barbarus* (64). Finally, analysis of blinking kinematics and testing of functional hypotheses were carried out in the larger of the two mudskipper species, *P. barbarus*.

### Animals.

The morphology of three species of goby was analyzed in this study: *P. barbarus*, *P. septemradiatus*, and *N. melanostomus*. Mudskippers were acquired through the pet trade and housed in a custom, semi-terrestrial aquarium where a 61 × 42-cm terrestrial section was connected by a small partially submerged ramp (ramp length: 48.25 cm at slope of ~18.5 degrees from the horizontal) to a 47 × 16-cm aquatic section that held brackish water (salinity: 5 ppt; pH: 7.75) at a maximum depth of 8 cm. The aquaria were kept in a room maintained at 25 °C and 33% relative humidity. Fishes were free to move between the terrestrial and aquatic sections. Housing and all experimental procedures were approved and carried out under Georgia Institute of Technology Institutional Animal Care and Use Committee guidelines (Protocol # A100272 to S.S.). In total, fifteen *P. barbarus* (standard lengths: 88 to 134 mm) were used in kinematic and functional analyses (*SI Appendix*, Table S5).

A round goby cadaver (N = 1) was donated by the Hale Lab at the University of Chicago. The specimen was originally collected as part of a previous study on fin sensory anatomy (81). The specimen was collected from Lake Michigan with a fishing rod, killed using a 0.5 g L^−1^ MS222 (tricaine methanesulfonate, Sigma-Aldrich, St Louis, MO, USA), and fixed in 4% paraformaldehyde for 3 d at 4 °C on a rocker. Round goby procedures were approved by The University of Chicago Institutional Animal Care and Use Committee guidelines (Protocol #71589 to Melina Hale).

### Microcomputed Tomography (µCT) Scanning.

*P. septemradiatus* (N = 1), *P. barbarus* (N = 1), and *N. melanostomus* (N = 1) were contrast stained and µCT scanned to analyze their cranial anatomy. Mudskippers were killed and fixed following the same procedure used for *N. melanostomus*. Once fixed, specimens were washed in phosphate-buffered saline (PBS) three times at room temperature, 10 min per wash. Then, they were stepped into a 20% by mass sucrose solution in PBS (steps of 5%, 10%, 15%, 20% sucrose solution, 1 h per step at room temperature). Samples were left in 20% sucrose overnight. Next, specimens were immersed in 5% solution (weight/volume) of phosphomolybdic acid (PMA) (PM Biomedicals, cat. no.: 51429-74-4) in PBS for 12 d. PMA is a contrast agent that allows for differentiation of a variety of tissues (e.g., muscle, bone, ligament, and nervous tissue) in μCT scans (82). Specimens in the staining solution were covered with foil to prevent photoreaction and placed on an orbital shaker at room temperature. After staining was complete, specimens were rinsed in PBS and scanned at The University of Chicago’s PaleoCT scanning facility with a GE Phoenix v|tome|x 240-kV/180-kV scanner with the 180-kV tube and no filters under the following parameters: *P. barbarus*—voxel size: 11.735 μm, voltage: 110 kV, current: 110 μA, timing: 1,000 ms, projections 2,000, frame averaging: 4, frames skipped: 1; *P. septemradiatus*—voxel size: 10.7 μm, voltage: 71 kV, current: 150 μA, timing: 1,000 ms, projections 1,200, frame averaging: 3, frames skipped: 1; *N. melanostomus*— voxel size: 24.25 μm, voltage: 105 kV, current: 100 μA, timing: 1,000 ms, projections 1,200, frame averaging: 3, frames skipped: 1.

### Histology.

Tissues from the eye, head, and body of *P. septemradiatus* were sectioned and stained with hematoxylin and eosin (H&E). Individuals (N = 2) were euthanized and fixed with methods described above. Once fixed, samples were washed in PBS for 5 min (3×) at room temperature and then stepped into 50%, 70%, 80%, 90%, 100% ethanol at 10 min per step. The eyes and associated soft tissues were then dissected from the cranium, and the lens was removed by an incision to the back of the eye. Tissues were left in 100% ethanol overnight and subsequently washed in xylene for 10 min at room temperature (2×), then into a 1:1 ratio of xylene and melted paraffin wax in a 60 °C water bath. Next, samples were washed three times in paraffin wax at 60 °C and positioned in a plastic mold (Fisher Scientific, cat. no.: 22-038-272) at room temperature to solidify. Once solid, the block was stored at −20 °C overnight. Paraffin blocks were sectioned on a Microm HM 330 microtome at 10 µm thickness. Sections were imaged on a Zeiss Axio Imager. M2 microscope with a mounted Axiocam 503 color camera (Carl Zeiss AG, Gena, Germany) under brightfield illumination. Images are “Extended Depth of Field” and generated from a z-stack of the section using the Zeiss proprietary software.

### Kinematics.

To characterize the trajectory and temporal dynamics of the eye and dermal cup during spontaneous blinks, we analyzed *P. barbarus*. Kinematics were collected from four individuals (*SI Appendix*, Table S5), three blinks per individual for a total of twelve blinks. Individuals were transported to a 20 × 20-cm custom tank made of 1/4-inch acrylic for filming. The tank floor was matted with polyester fiber wetted with tank water to provide traction and moisture. Filming was conducted in the same room as where the fish were housed and, therefore, under the same environmental conditions. After being placed in the filming tank, animals were allowed to acclimate for 20 min and then filmed continuously at 100 frames per second using three synchronized Blackfly S USB 3.0 machine vision cameras (BFS-U3-13Y3C, FLIR Systems, Inc., North Billerica, MA, USA) at a resolution of 1,280 × 1,024, exposure of 6,002, and gain of 10. Two cameras were placed at the height of mudskipper eyes, parallel to the ground and orthogonal to each other. The third camera was mounted orthogonally to the first two cameras, filming the mudskippers from above. Cameras were calibrated using a checkerboard composed of 4.8-mm squares arranged four rows by six columns. Videos were calibrated and digitized through XMALab version 1.5.1 (83). Calibration relied on the digitization of at least 40 nonsequential frames of the calibration object and 100,000 optimization iterations. We then digitized the apex of the eye and the dermal cup of a single eye for every frame for the entirety of a single blink in at least two views. A body coordinate system was established through additional landmarks placed at the apex of the contralateral eye, the rostral tip of the snout, the rostral articulation of the dorsal fin, and the dorsorostral articulation of the left and right pectoral fins.

From the exported time-series of the landmarks, the position of the eye and the dermal cup was tracked over time. Upon initial inspection, we found that significant movement of the eye and dermal cup took place only in the dorsal-ventral direction. Subsequent analyses, therefore, focused on the kinematics in the lateral 2D plane. Velocity and acceleration of the eye and dermal cup were calculated from the position data. Blinks were normalized for their length and divided into two phases: eye depression (downstroke) and eye elevation (upstroke). The start of downstroke was when eye movement was first detected. The end of the downstroke was when downward motion ceased for at least one frame. The upstroke began immediately following the end of the downstroke, and it ended when the eye returned to its initial point or reached a velocity of 0.0 cm/s; in some instances, the eye did not fully return to the same elevation as the start of the blink despite the cessation of movement. Exported time-series landmark data were analyzed in MATLAB (version R2018b–9.5.0.944444).

The close-up images of the Indian mudskipper, *P. septemradiatus*, in Fig. 2*A* were captured using a BK PLUS lab system by Dun, Inc. with a 20× microscope objective on a Canon DSLR camera.

### Evaporation Studies.

To analyze whether blink rate is related to drying of the eye, we recorded *P. barbarus* under typical environmental conditions and under a high-evaporation condition. Individuals (N = 6; *SI Appendix*, Table S5) were transferred to a 20- × 20-cm custom tank made of 0.25-inch acrylic outfitted with evenly spaced 0.25-inch diameter holes around the perimeter of the tank base. A custom lid equipped with two computer fans (upHere 12bk3-3, Guangdong, China) was used to induce air flow. The fans were driven by a 12V power source (Flexzion 305D, Los Angeles, CA) and drew air upward, through the holes at the tank base and out the top of the tank. We estimated evaporation rate using the US EPA Evaporation Equation: E = (7.41*A*P*(0.44V)^0.78^)/(T+459.67). The estimate considers surface area (A), water vapor pressure at ambient temperature (P), ambient temperature (T), and ambient air velocity (V) (84, 85). By varying ambient air velocity (~75 times higher when the fan is on), we calculated that the high-evaporation condition has an evaporation rate that is 30 times greater than the control conditions.

For each trial, individuals were transferred to the experimental tank and allowed to acclimate for 30 min to ensure the temporary handling did not influence stress or blink frequency. In three individuals, the high-evaporation condition was presented first, for 1 h. Then, the fans were turned off for 30 min for acclimatization, and then they were recorded with the fans turned off, for a second hour. In three other individuals, the order of the experimental condition (i.e., fans off versus on) was reversed. We cataloged the timestamp of each blink for both the left and right eyes, and we calculated the interblink interval of each eye. Upon review of the recordings, we also observed that mudskippers roll around the long axis of their body on a regular basis. Therefore, we cataloged the timestamp of rolls to either the left or right side of the body and calculated IRI for each direction of rolling.

### Mechanically Stimulated Blinking.

To assess the mechanosensory capabilities of the corneal surface and determine if mechanical stimulation of the corneal surface resulted in a blink, we recorded the behavioral response of *P. barbarus* to gentle mechanical stimulation. The lateral corneal surface of the eye was lightly tapped with a custom-built capacitive sensor, which was made by integrating a Q-Tip cotton swab (Unilever, Englewood Cliffs, USA) with conductive wire. The sensor was controlled by an Arduino UNO (Arduino, Monza, Italy) that activated an LED light when the sensor was in direct contact with a capacitive surface, such as the mudskipper eye. The eye and LED light were both in view and recorded by a Fastec TS4 camera (Fastec, San Diego, CA) at 1,000 frames per second. The lag time between mechanical stimulation and the blink was calculated as the time between the onset of the LED light and the initiation of the blink. We conducted this experiment in five individuals of *P. barbarus* (*SI Appendix*, Table S5) and recorded at least 10 mechanically stimulated blinks from each individual. Trials ended when the individual left the arena. The response did not show any signs of habituation. This experiment was conducted in the same tank and under the same environmental conditions as the kinematic recordings.

### Cleaning of the Eye.

To analyze the cleaning capacity of blinking in *P. barbarus*, we analyzed their ability to remove debris from the eye in a single blink. Cleaning capacity was measured in five individuals of *P. barbarus* (*SI Appendix*, Table S5); at least eight blinks per single eye per individual were recorded. Individuals were transferred to the same tank used for kinematic experiments, maintained under the same environmental conditions, and allowed to acclimate for 30 min prior to the start of the experiment. Individuals were filmed using a Fastec IL5 (Fastec, San Diego, CA) high-speed camera at 1,000 FPS. Dry brine shrimp eggs (Brine Shrimp Direct, Ogden, UT, USA) were then sprinkled evenly onto the cornea. The number of eggs ranged from 1 to 43 with a mean ± SD of 16 ± 9 eggs applied per trial. The number of eggs on the eye were counted before and after each blink to calculate the percentage of eggs removed by a single blink.

### Statistical Analysis.

All statistical analyses were performed in RStudio (v. 1.1.383) using R (v. 4.0.2). Two tailed, type 2 Student *t* tests were conducted to test for significant differences between kinematic variables measured during the first and second halves of the blink duration, the experimental conditions (control versus high-evaporation rate) for the interblink interval and IRIs, and spontaneous and mechanically stimulated blinks, which focused on both the duration and speed of downstroke.

## Supplementary Material

Appendix 01 (PDF)

Movie S1. Blink of the Indian mudskipper *P. septemradiatus* at full speed

Movie S2. Blink of the African mudskipper *P. barbarus* at full speed

Movie S3. Blink of the African mudskipper *P. barbarus*, slowed by 20x

Movie S4. Volumetric rendering of μCT data of the African mudskipper *P. barbarus* showing extraocular muscles

Movie S5. Digital cross sections of μCT data of the African mudskipper *P. barbarus*

Movie S6. Roll of the African mudskipper *P. barbarus*, slowed by 5x

Movie S7. Exemplar blink showing cleaning, slowed by 5x

Movie S8. Exemplar mechanically stimulated blink, slowed by 10x

## Data Availability

CT data have been deposited in MorphoSource (Project ID: 000489938; specimens 10.17602/M2/M494574, 10.17602/M2/M494706, 10.17602/M2/M494701) (86). Video data have been deposited to Dryad (doi:10.5061/dryad.sj3tx968w) (87). All study data are included in the article and/or supporting information.
