# Supplementary material for: The origin of blinking in both mudskippers and tetrapods is linked to life on land
Source: Proc Natl Acad Sci U S A. 2023 Apr 24;120(18):e2220404120. doi: 10.1073/pnas.2220404120 (PMC10160996; doi:10.1073/pnas.2220404120)
Supplement: Supplementary file 1 — Appendix 01 (PDF) [file pnas.2220404120.sapp.pdf]

Supplementary Materials for

The origin of blinking in both mudskippers and tetrapods is linked to life on land

Brett R. Aiello, M. Saad Bhamla, Jeff Gau, John G.L. Morris, Kenji Bomar, Shashwati da Cunha, Harrison Fu, Julia Laws, Hajime Minoguchi, Manognya Sripathi, Kendra Washington, Gabriella Wong, Neil H. Shubin, Simon Sponberg*, Thomas A. Stewart*

*Corresponding authors. nshubin@uchicago.edu, sponberg@gatech.edu, tomstewart@psu.edu

This PDF file includes:

Fig. S1
Tables S1 to S5
Legends for movies S1 to S8

Other Supplementary Materials for this manuscript include the following:

Movies S1 to S8

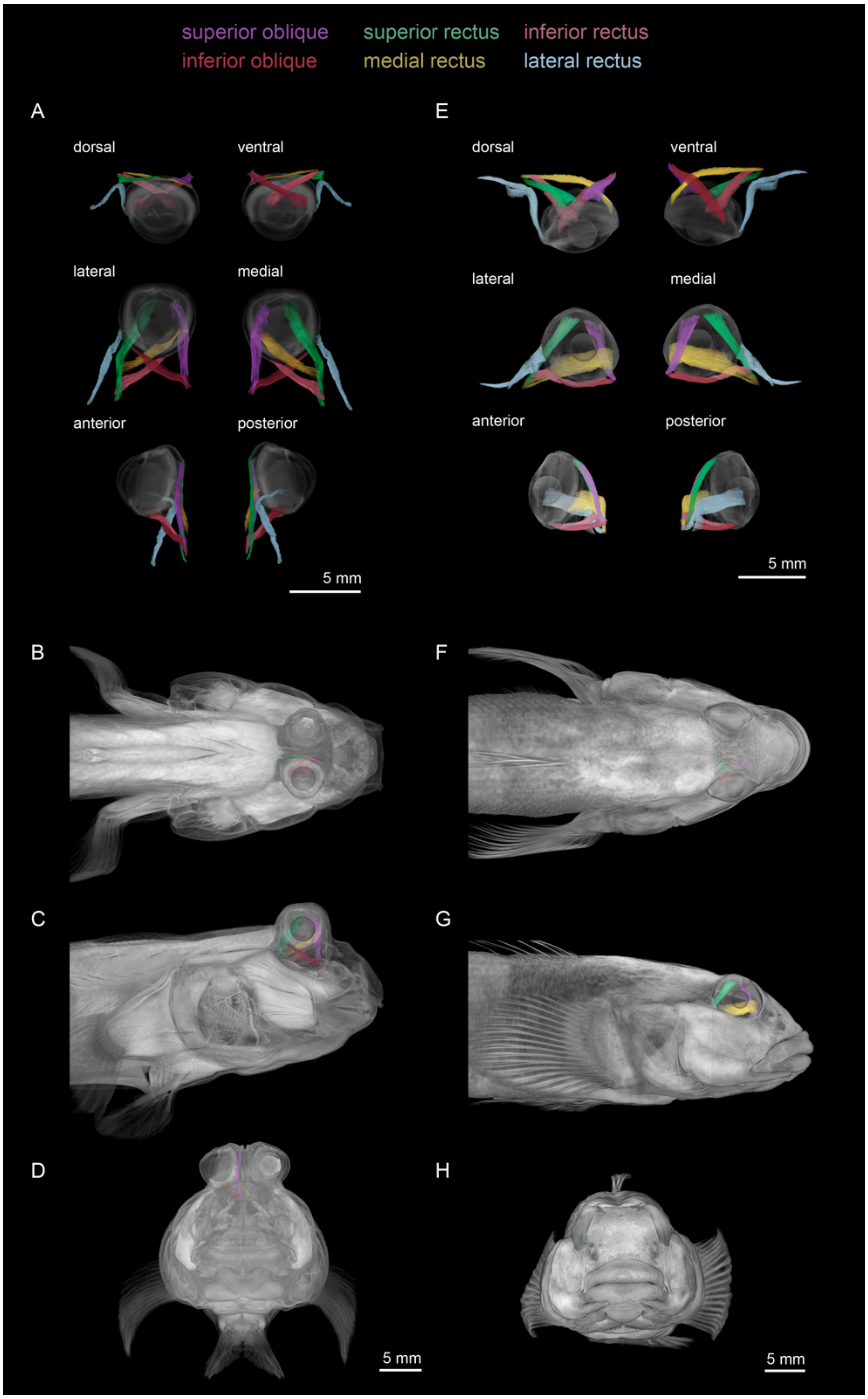

Fig. S1. Volumetric rendering of contrast-stained Indian mudskipper and round goby. *P. septemradiatus* **(A)** eye and six extraocular muscles, and anterior portion of the body in **(B)** dorsal, **(C)** lateral, and **(D)** anterior views. *N. melanostomus* **(E)** eye and six extraocular muscles, and anterior portion of the body in **(F)** dorsal, **(G)** lateral, and **(H)** anterior views.

Table S1

Individual	Blink #	Blink dur. (s)	Down-stroke dur. (s)	Down-stroke dur. (% blink cyc.)	Up-stroke dur. (s)	Start of DC move-ment (% blink cyc.)	Start of DC-eye inter-action (% blink cyc.)	End of DC-eye inter-action (% blink cyc.)	Peak eye velocity, down-stroke (cm s^{-1})	Peak eye velocity, up-stroke (cm s^{-1})	Amplitude of eye depression (cm)
MS1	1	0.51	0.20	39.22	0.31	3.92	11.76	70.59	-3.59	1.83	-0.33
MS1	2	0.57	0.21	36.84	0.36	3.51	10.53	66.67	-3.71	1.78	-0.29
MS1	3	0.54	0.21	38.89	0.33	1.85	14.81	70.37	-3.94	2.64	-0.38
	mean	0.54	0.21	38.32	0.33	3.09	12.37	69.21	-3.75	2.08	-0.34
	stdev	0.03	0.01	1.29	0.03	1.10	2.21	2.20	0.18	0.48	0.04
MS2	1	0.49	0.18	36.73	0.31	2.04	12.24	73.47	-3.50	2.42	-0.33
MS2	2	0.53	0.17	32.08	0.36	1.89	11.32	69.81	-3.64	1.21	-0.27
MS2	3	0.48	0.16	33.33	0.32	2.08	10.42	77.08	-3.68	1.64	-0.27
	mean	0.50	0.17	34.05	0.33	2.00	11.33	73.45	-3.61	1.75	-0.29
	stdev	0.03	0.01	2.41	0.03	0.10	0.91	3.64	0.09	0.61	0.03
MS3	1	0.70	0.26	37.14	0.44	2.86	7.14	71.43	-3.31	1.24	-0.34
MS3	2	0.64	0.2	31.25	0.44	3.13	10.94	64.06	-3.17	1.14	-0.31
MS3	3	0.68	0.27	39.71	0.41	1.47	7.35	82.35	-4.10	1.49	-0.34
	mean	0.67	0.24	36.03	0.43	2.48	8.48	72.61	-3.53	1.29	-0.33
	stdev	0.03	0.00	4.34	0.02	0.89	2.13	9.20	0.50	0.18	0.02
MS4	1	0.52	0.17	32.69	0.35	1.92	9.62	75.00	-3.10	1.46	-0.26
MS4	2	0.52	0.17	32.69	0.35	1.92	9.62	71.15	-3.02	1.77	-0.32
MS4	3	0.54	0.17	31.48	0.37	1.85	11.11	74.07	-3.30	1.87	-0.30
	mean	0.53	0.17	32.29	0.36	1.90	10.11	73.41	-3.14	1.71	-0.29
	stdev	0.01	0.00	0.70	0.01	0.04	0.86	2.01	0.15	0.22	0.03
Summary	mean	0.56	0.20	35.17	0.36	2.37	10.57	72.17	-3.51	1.71	-0.31
	stdev	0.07	0.04	3.22	0.05	0.78	2.07	4.77	0.33	0.46	0.04

Blinking kinematics data correspond to the graphs presented in Fig. 2B. In the column headers, ‘dermal cup’ is abbreviated as DC.

Table S2

	Inter-blink interval (IBI)					Inter-roll interval (IRI)				
Individual	Control IBI	# of blinks	High evap. IBI	# of blinks	*p* value	Control IRI	# of rolls	High evap. IRI	# of rolls	*p* value
MS1	99.5 ± 64.5	68	30.7 ± 31.9	231	4.74 x10^{-27}	--	0	171.6 ± 227.0	40	--
MS2	202.4 ± 114.5	32	28.3 ± 36.4	249	3.01 x10^{-48}	--	1	71.5 ± 56.6	96	--
MS3	421.0 ± 237.9	9	135.3 ± 217.3	46	9.99 x10^{-04}	--	0	135.3 ± 217.3	94	--
MS4	151.6 ± 154.2	44	46.9 ± 49.2	139	7.61 x10^{-11}	744.5 ± 191.5	4	152.8 ± 138.4	45	6.18 x10^{-10}
MS5	121.0 ± 84.8	68	29.1 ± 24.2	243	1.89 x10^{-27}	287.7 ± 288.0	9	303.4 ± 198.5	20	0.87
MS6	97.6 ± 71.4	71	30.7 ± 29.9	227	9.68 x10^{-25}	428.1 ± 214.7	14	285.8 ± 189.2	24	0.04
Summary	128.9 ± 120.5	292	36.1 ± 58.9	1135	7.1345 x10^{-70}	428.2 ± 279.9	28	127.1 ± 147.5	319	4.90 x10^{-19}

Inter-blink and inter-roll intervals in control and high evaporation conditions. The *p* values are calculated from a double sided, type 2 t-test.

Table S3

Individual	Eye side	% Cleaned
MS1	R	99 ± 1
MS2	L	96 ± 8
MS3	L	96 ± 7
MS4	L	97 ± 9
MS5	R	99 ± 3
Summary		97 ± 7

The cleaning efficiency of a single blink, which was calculated as the percentage of brine shrimp eggs removed from the cornea in one blink.

Table S4

Individual	Lag time (ms)	Eye depression duration (ms)
MS1	26 ± 5	77 ± 22
MS2	28 ± 9	101 ± 33
MS3	28 ± 6	94 ± 31
MS4	25 ± 6	94 ± 28
MS5	31 ± 6	96 ± 28
Summary	28 ± 7	93 ± 30

Measurement of lag time and eye depression duration in mechanically stimulated blinks. Summary values represent mean and standard deviation.

Table S5

Experiment	Individual	Eye diameter (mm)	Standard length (mm)	Total length (mm)
Eye kinematics	MS1	5.09	93.30	109.25
	MS2	4.97	88.57	103.89
	MS3	5.05	97.95	110.77
	MS4	4.94	114.68	124.42
Evaporation	MS1	6.09	110.81	126.24
	MS2	6.80	110.89	117.17
	MS3	5.53	90.94	99.00
	MS4	6.37	107.44	117.93
	MS5	6.86	123.87	146.96
	MS6	7.02	133.89	153.34
Mechanical stimuli and cleaning	MS1	6.49	124.29	135.07
	MS2	6.06	126.42	142.45
	MS3	6.26	117.25	137.69
	MS4	6.12	124.43	130.68
	MS5	5.49	102.28	112.35

Morphometrics of individuals used in the functional analyses. A total of 15 individuals were analyzed.

Movie S1
Blink of the Indian mudskipper *P. septemradiatus* at full speed

Movie S2
Blink of the African mudskipper *P. barbarus* at full speed

Movie S3
Blink of the African mudskipper *P. barbarus*, slowed by 20x

Movie S4
Volumetric rendering of µCT data of the African mudskipper *P. barbarus* showing extraocular muscles

Movie S5
Digital cross sections of µCT data of the African mudskipper *P. barbarus*

Movie S6
Roll of the African mudskipper *P. barbarus*, slowed by 5x

Movie S7
Exemplar blink showing cleaning, slowed by 5x

Movie S8
Exemplar mechanically stimulated blink, slowed by 10x
